# Response of conventional chondrosarcoma to gemcitabine alone: a case report

**DOI:** 10.1186/s13569-015-0025-z

**Published:** 2015-03-15

**Authors:** Salvatore Provenzano, Nadia Hindi, Carlo Morosi, Mara Ghilardi, Paola Collini, Paolo G Casali, Silvia Stacchiotti

**Affiliations:** Adult mesenchymal tumour & Rare cancer Medical Oncology Unit, Cancer Medicine Department, Fondazione IRCCS Istituto Nazionale Tumori, via G. Venezian, 1 I-20133 Milan, Italy; Department of Radiology, Fondazione IRCCS Istituto Nazionale Tumori, Milan, Italy; Medical Oncology Unit, Ospedale di Treviglio, Azienda Ospedaliera Treviglio, Treviglio, (BG) Italy; Department of Diagnostic Pathology and Laboratory Medicine, Fondazione IRCCS Istituto Nazionale Tumori, Milan, Italy

**Keywords:** Sarcoma, Bone sarcoma, Chondrosarcoma, Gemcitabine, Chemotherapy

## Abstract

Conventional skeletal chondrosarcoma is a bone neoplasm, which is poorly sensitive to anthracyclines-based chemotherapy. We report on an 18-month-long tumour response to gemcitabine as single agent in a young patient with an advanced secondary peripheral conventional chondrosarcoma, previously treated unsuccessfully with anthracyclines, ifosfamide, platinum, etoposide.

## Background

The skeletal chondrosarcoma family represents a heterogeneous group of malignant bone mesenchymal tumours characterised by the production of a chondroid matrix. They are the third bone sarcoma in incidence, and the most frequent in adults. There are three main subtypes: conventional, mesenchymal, and clear-cell. A “dedifferentiated” chondrosarcoma develops in 10-15% of conventional chondrosarcomas, while mesenchymal chondrosarcoma is a high-grade, aggressive neoplasm with a natural history and chemosensitivity that might be close to Ewing sarcoma, and clear-cell chondrosarcoma is a low-grade variant [1].

In conventional chondrosarcoma (cCS), the histological malignancy grade is the main prognostic factor [2]. Grade 1 cCS are characterised by a very low metastatic potential, and some authors have quite recently suggested a re-classification of these types as “atypical cartilaginous tumours” [1]. Grade 2 and 3 cCS are marked by a higher metastatic potential, with a 10-year survival of 64-86% and 29-55% respectively [3,4].

CCSs are also categorised according to their location in the bone: a central chondrosarcoma onsets in the medullary cavity, a small percentage of them from a pre-existing benign lesion known as enchondroma, while a peripheral variant arises from the surface of the bone, as a result of malignant progression of a pre-existing benign (solitary or hereditary) osteochondroma.

Surgery is the mainstay of the treatment of localized disease. While curettage is acceptable for grade 1 cCS, wide excision is usually required for higher grade cCS, with the exception of skull base cCS which may be treated with radiotherapy. In particular, hadrons can play an important role in the management of skull base cCS, and very good outcomes are reported [5].

In surgically treated patients, the benefit of adding radiotherapy and chemotherapy remains unclear, due to a lack of prospective trials. Adjuvant radiotherapy and/or chemotherapy may be proposed to high-risk patients in conditions of uncertainty. When cCS is advanced, and a medical therapy is the only option, regimens commonly used in other bone sarcomas are employed [6]. Traditionally, chemotherapy has been considered poorly effective [7], but the low number of cases and the inclusion in available series of conventional (both central and peripheral), dedifferentiated, mesenchymal, clear-cell histotypes does not help to understand the actual chemo-responsiveness of the disease. Recently, responses to gemcitabine in combination with docetaxel have been reported in advanced chondrosarcomas [8].

Hereby, we describe the case of a young woman with a metastatic, pretreated cCS treated with gemcitabine as a single agent, after failing to anthracyclines, ifosfamide, cisplatin, etoposide.

## Case presentation

### Patient characteristics and medical history

**Figure 1:**
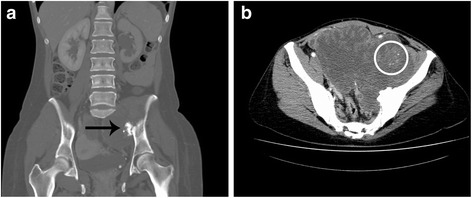
**Contrast-enhanced CT scan performed at the time of diagnosis in December 2009. (a)** Presence of a large mass arising from an osteochondroma (arrow) of the left iliac bone (coronal plane, bone window, venous phase); **(b)** the primary tumour appears as a poli-lobulated mass extending within the pelvis, characterised by an irregular, peripheral contrast enhancement and scattered calcification islets (circle) (axial plane, abdomen window, arterial phase).

**Figure 2:**
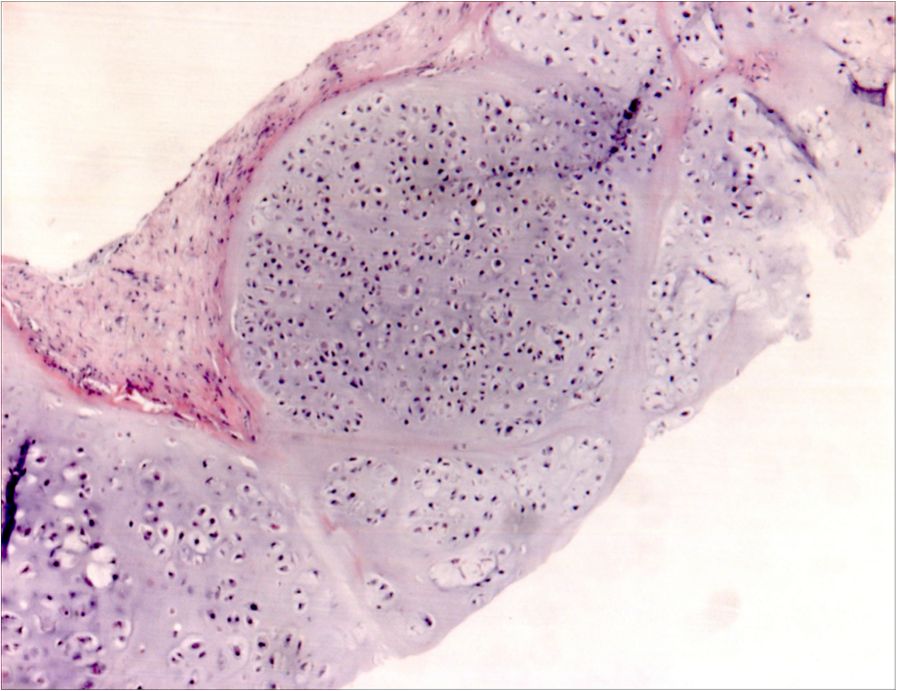
**Tru-cut biopsy of the pelvic, primary tumour, performed in December 2009.** Histopathological examination (HE x5, inset x10): fibrous tissue with nests of cartilaginous proliferation with hypercellularity and variation in cellular size and shape, in a focally myxoid matrix. Final diagnosis was G2 peripheral conventional chondrosarcoma. Radiologic features were not consistent with the presence of dedifferentiated areas thus supporting the final diagnosis of a conventional chondrosarcoma.

In December 2009, a 38-year old woman, in good general conditions, was diagnosed a 17-cm large mass arising from an osteochondroma of the left iliac bone (Figure 1). Diagnostic biopsy revealed grade 2 secondary peripheral cCS (Figure 2). Staging for distant metastases was negative and no other osteochondromas were found. No familial history of osteochondromatosis was referred.

Front-line surgery was ruled out because of the extent of the disease, the major blood vessels and nerves being involved. In February 2010, chemotherapy with full-dose doxorubicin plus ifosfamide was administered for 3 cycles, but tumour progression ensued. In April 2010, definitive external beam radiotherapy (total dose 72 Gy) achieved a minor dimensional response and symptom control.

**Figure 3:**
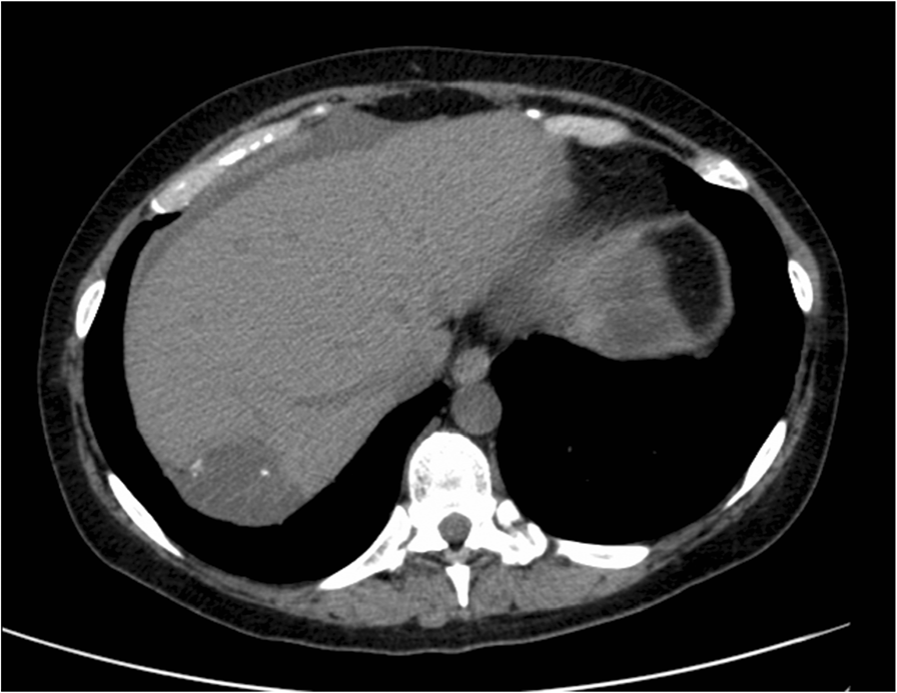
**CT scan without contrast of the liver at the time of the first hepatic progression, showing a single metastasis, characterised by pronounced hypodensity and calcification islets (axial plane, abdomen window).**

In July 2012, the disease progressed locally and gave a single liver metastasis, confirmed on biopsy (Figure 3). Chemotherapy with 14-day prolonged infusion of high-dose ifosfamide was administered for one cycle but had to be withdrawn due to neurotoxicity. Chemotherapy with cisplatin and etoposide for 2 cycles was given, with progression of the disease.

**Figure 4:**
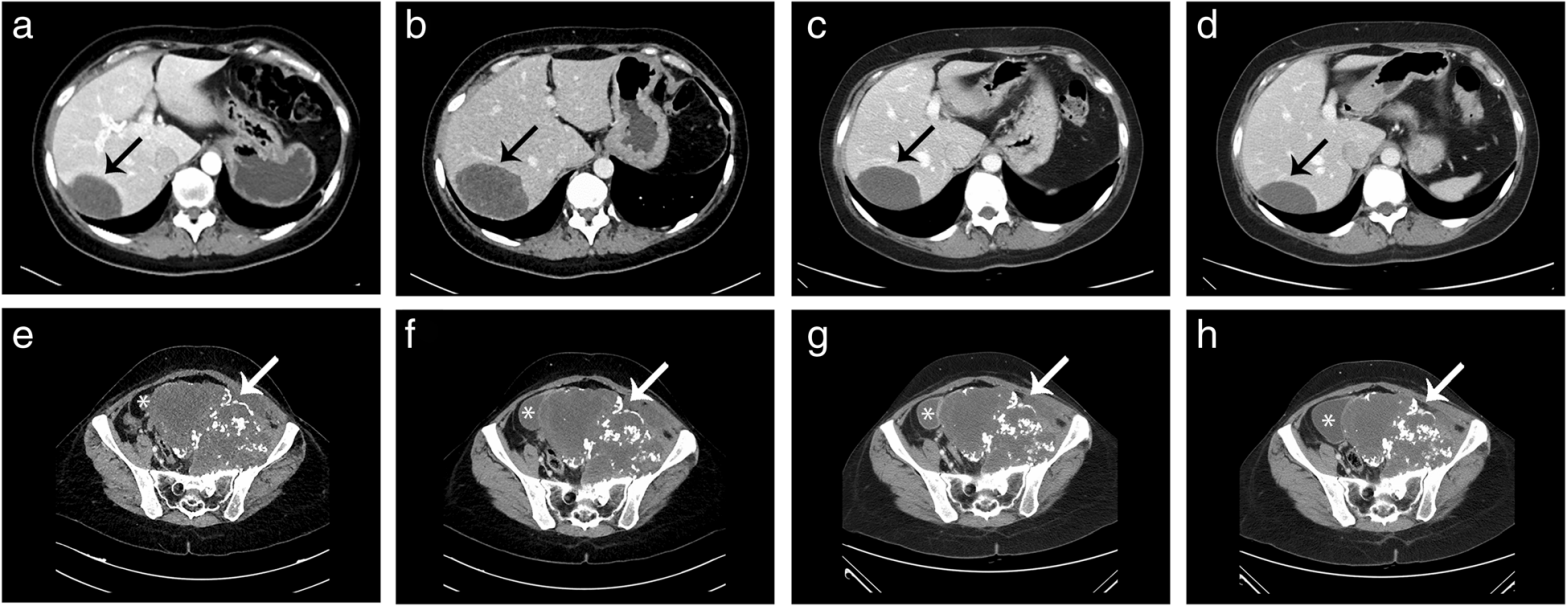
**Contrast-enhanced CT scan of the liver and the pelvis (axial plane, abdomen window, venous phase).** The progressive hepatic metastasis (black arrow) before **(a)** and after **(b)** chemotherapy with cisplatin/etoposide, then a RECIST response after 4 **(c)** and 9 **(d)** cycles of gemcitabine. Pelvis reports **(e-h)** appear stable (white arrow: primary tumour; asterisk: urinary bladder).

In December 2012, in the lack of alternative options, a fourth-line chemotherapy was started with gemcitabine (1,000 mg/sqm on day 1,8,15, every 28 days, administered intravenously in 30’). By RECIST the disease looked stable with regard to the pelvic, primary lesion, while a partial response of the liver lesion was observed (Figure 4). A significant improvement of symptoms, i.e. pain and walking impairment due to compression of the left femoral nerve by the primary tumour, was achieved, and progressively led to withdraw analgesics, i.e., fentanyl TTS 50 mcg/h and pregabalin 300 mg twice/day.

Neutropenia G1-G2 was recorded as the only side effect to gemcitabine.

In September 2013, chemotherapy was interrupted after 9 cycles as for patient request. Tumour response was maintained until February 2014 when, after a-14 month progression-free survival, a disease progression was observed to the liver and to the abdomen, with the new appearance of multiple peritoneal metastases. In May 2014, gemcitabine was restarted, with progression at the following evaluation in September 2014.

## Discussion

This is the first report of a response to gemcitabine as single agent, in a patient with a heavily pre-treated advanced cCS. The response was marked by a regression of the tumour and symptomatic improvement. While high-grade chondrosarcoma subtypes (mesenchymal and dedifferentiated) can be sensitive to chemotherapy [9], cCS is usually described as a chemo-refractory disease [10]. Indeed, in high-grade chondrosarcomas responses to chemotherapy have been reported to anthracycline-based chemotherapy combinations, and they are usually treated with regimens commonly used in high-grade osteosarcomas, also including cisplatin [7,11-13].

Trials with targeted-therapy have not proven efficacy so far. The initial enthusiasm on hedgehog inhibitors coming from pre-clinical studies, on the basis of a strong activation of the hedgehog pathway in chondrosarcomas, has been cut down after the results of a phase 2 trial in which no objective responses were observed [14].

Gemcitabine is a nucleoside analogue of deoxycytidine, which metabolizes in cell to its active metabolites gemcitabine diphosphate (dFdCDP) and gemcitabine triphosphate (dFdCTP) [15]. Gemcitabine, both as single agent or in combination with taxanes, is known to be active in soft tissue sarcomas, especially leiomyosarcoma and angiosarcoma [16-19]. As regards bone sarcomas, some small studies reported objective responses with gemcitabine and docetaxel in osteosarcoma and Ewing’s sarcoma [8,20,21]. Some responses have been reported with gemcitabine-based regimens in the treatment of chondrosarcoma, yet the experience with this combination of drugs is very limited (data summarised in Table 1).

**Table 1:** **Summary of available studies reporting on gemcitabine in the treatment of chondrosarcomas (all histotypes)**

	***Type of study***	***Regimen***	***Patients (n)***	***Best response (RECIST)***
Merimsky et al., 2000 [25]	Prospective	Gemcitabine alone	3	2 SD, 1 PD
Fox et al., 2012 [8]	Prospective	Gemcitabine/docetaxel	25	2 PR, 14 SD, 9 PD
Italiano et al., 2013 [7]	Retrospective	Gemcitabine-based combinations	n/a	3 PR

Legend. RECIST: response evaluation criteria in solid tumour; SD: stable disease; PD: progressive disease; PR: partial response; n/a: not available.

Though a single case, this is the first report of responsiveness of cCS to gemcitabine alone. This patient was experiencing a progressive disease with worsening symptoms before gemcitabine. After starting gemcitabine, pain and walking impairment quickly improved. Tumour response was a RECIST partial response to the site of liver metastasis, and a minor tumour shrinkage of the primary, pretreated with RT, lesion. Response was long lasting. Interestingly, this patient had not responded to any of the drugs used previously.

To be noted, the patient described in this case report carried a secondary peripheral chondrosarcoma. Differently from central variant, secondary peripheral chondrosarcomas have distinct molecular features. In fact, solitary osteochondromas are characterised by homozygous deletion of EXT-1 (exostosin) gene, which is involved in heparan sulfate biosynthesis. This alteration may lead to a dysregulation of downstream signalling pathways and, eventually, progression to secondary peripheral chondrosarcoma [22,23]. On this basis, different targets as compared to primary central chondrosarcoma have been identified [24] in secondary peripheral chondrosarcoma and a different chemosensitivity cannot be excluded. Even if, at least of our knowledge, there are no data to sustain at present a higher sensitivity to gemcitabine in presence of an EXT-1 deletion and the available reports on the activity of gemcitabine/docetaxel in chondrosarcoma do not go into details on the chondrosarcoma subtype, this cannot be ruled out.

## Conclusion

We add to anecdotal evidence that gemcitabine-based chemotherapy may be active in cCS. This needs to be confirmed through prospective studies targeted to cCS, without the confounding factor of other histotypes. Whether the combination of docetaxel and gemcitabine can be more active than gemcitabine single agent in cCS is left to understand, considering the extra-toxicity implied by the combination in comparison to single-agent gemcitabine.

## Consent

Written informed consent was obtained from the patient for publication of this Case Report and any accompanying images. A copy of the written consent is available for review by the Editor-in-Chief of this journal.
